# Beyond macrophages: FIPV tropism includes T and B lymphocytes

**DOI:** 10.1016/j.vetmic.2025.110864

**Published:** 2025-12-31

**Authors:** Aadhavan Balakumar, Patrawin Wanakumjorn, Kazuto Kimura, Ehren McLarty, Katherine Farrell, Terza Brostoff, Jully Pires, Tamar Cohen-Davidyan, Jennifer M. Cassano, Brian Murphy, Krystle Reagan, Amir Kol

**Affiliations:** a Pathology, Microbiology and Immunology, School of Veterinary Medicine, University of California, Davis, CA, USA; b Veterinary Surgical and Radiological Sciences, School of Veterinary Medicine, University of California, Davis, CA, USA; c The Veterinary Center for Clinical Trials, School of Veterinary Medicine, University of California, Davis, CA, USA; d The Regenerative Medicine Laboratory, Veterinary Institute for Regenerative Cures, School of Veterinary Medicine, University of California, Davis, CA, USA; e Veterinary Medicine and Epidemiology, School of Veterinary Medicine, University of California, Davis, CA, USA

**Keywords:** Viral tropism, Feline infectious peritonitis virus, Lymphocyte infection

## Abstract

If untreated, feline infectious peritonitis (FIP) is a fatal disease that is caused by feline infectious peritonitis virus (FIPV), a virulent biotype of feline coronavirus (FCoV) that disseminates broadly and triggers severe systemic inflammation. While the prevailing model holds that FIPV selectively infects monocytes/macrophages, the full range of susceptible cell types and the mechanisms of immune cell invasion remain poorly defined. Here, we applied single-cell RNA sequencing, multiplex immunofluorescence, and *in situ* hybridization to mesenteric lymph node aspirates and formalin fixed and paraffin embedded lymph node tissues from cats with naturally occurring effusive FIP. We identified FIPV RNA and nucleocapsid protein in T and B lymphocytes and myeloid cells, and subgenomic viral RNA in T cells, demonstrating cell entry and viral genomic replication across multiple immune compartments. Rare FIPV RNA–positive lymphocytes persisted after antiviral treatment cessation and resolution of clinical signs. These findings revise current models of FIPV pathogenesis and reveal new insights into coronavirus-driven immune dysregulation, viral persistence, and relapse. Our study highlights the utility of FIP as a naturally occurring animal model for exploring adaptive immune cell infection in coronavirus diseases, providing a translational platform for understanding virus–host interactions that drive chronic or relapsing immunopathology.

## 1. Introduction

Feline infectious peritonitis (FIP) is a progressive and fatal disease of domestic cats if left untreated that results from one of several described virulent mutations/deletions of feline coronavirus (FCoV), enabling systemic spread and immune-mediated pathology (Weiss and Scott, 1981; Sparkes et al., 1991; Hartmann, 2005; Pedersen, 2009; Riemer et al., 2016; Kennedy, 2020; Tasker et al., 2023). While the prevailing model holds that the mutated form of FCoV, feline infectious peritonitis virus (FIPV), selectively infects monocytes/macrophages, the full range of susceptible cell types and the mechanisms of immune cell invasion remain poorly defined (Hayashi et al., 1978; Hok, 1990; Tammer et al., 1995; Rottier et al., 2005). FCoV and FIPV exist in two serotypes most commonly genotypically distinguished by differences in their spike (S) protein sequences and receptor usage (Cook et al., 2022). Serotype 2 FCoV arose through historic recombination event with canine coronavirus and utilizes feline aminopeptidase N (fAPN) as its cellular receptor, enabling studies of viral entry and infection mechanisms *in vitro (*Herrewegh et al., 1998; Hohdatsu et al., 1998). In contrast, serotype 1 FCoV, which accounts for the vast majority of naturally occurring FIP cases (Benetka et al., 2004; Kummrow et al., 2005; Li et al., 2019), does not utilize fAPN and its host cell receptor(s) remain unknown (Hohdatsu et al., 1998). Recent advances, including the development of molecular tools compatible with feline tissues and the emergence of antiviral therapies (Murphy et al., 2018; Taylor et al., 2023), have positioned FIP as a powerful comparative model for studying coronavirus immunopathogenesis in a natural host.

FCoV is endemic in multi-cat environments and is typically associated with subclinical or mild enteric infection. In a subset of cases, viral mutations, most notably in the spike gene, enable systemic spread and replication in monocytes and macrophages, resulting in the emergence of FIPV (Tusell et al., 2007; Tekes et al., 2010; Chang et al., 2012; Bank-Wolf et al., 2014; Decaro et al., 2021). The ensuing disease is characterized by pyogranulomatous perivasculitis, fibrinous serositis, and multiple organ injury (Kennedy, 2020; Tasker et al., 2023; Murphy et al., 2024a). FIP presents in two main forms: effusive (wet) form with cavitary fluid accumulation, and non-effusive (dry) disease characterized by granulomatous inflammation affecting multiple organs such as the brain, eye, liver, kidneys, intestine, and lymph nodes (Kennedy, 2020; Tasker et al., 2023). Although macrophage tropism is considered central to FIP pathogenesis, the mechanisms that underlie systemic inflammation, lymphoid depletion, and tissue-specific pathology remain incompletely understood.

FIP shares striking clinical and immunological features with severe human coronavirus infections, including severe COVID-19, multisystem inflammatory syndrome in children (MIS-C), and post-acute sequelae of SARS-CoV-2 infection (Long COVID). These syndromes are characterized by cytokine dysregulation, immunopathology, and persistent or relapsing disease (Taylor et al., 2023; Murphy et al., 2024a; Kipar et al., 1998; Zwicklbauer et al., 2023; Trsar et al., 2025; Wanakumjorn et al., 2025). However, the cellular basis of these outcomes, particularly the roles of viral persistence and immune cell infection, remains poorly understood in humans due to limited access to infected tissues. In contrast, FIP presents a unique opportunity to study coronavirus–host interactions directly within lymphoid organs of a naturally infected large animal host.

To date, the prevailing model of FIP pathogenesis has emphasized monocyte and macrophage infection as the primary mechanism of viral dissemination and immune activation (Hayashi et al., 1978; Hok, 1990; Tammer et al., 1995; Rottier et al., 2005; Kipar et al., 1998; Ward, 1970; Pedersen, 1976; Stoddart and Scott, 1989; Martinez and Weiss, 1993; Dewerchin et al., 2005; Belouzard et al., 2012; Cham et al., 2017; Shirato et al., 2018). Yet, this model does not fully explain the immunological collapse and clinical relapse observed in some treated cats (Gokalsing et al., 2025). Moreover, there is growing interest in whether coronaviruses, including severe acute respiratory syndrome (SARS)-CoV-2 and Middle East respiratory syndrome (MERS)-CoV, can infect lymphocytes and other adaptive immune cell subsets (Pontelli et al., 2022; Zhou et al., 2014; Coleman et al., 2017; Brunetti et al., 2023). Definitive evidence of such cellular permissiveness in human patients remains scarce.

In this study, we used an integrative spatial and transcriptomic approach to investigate the cellular tropism of FIPV in mesenteric lymph nodes from cats with naturally occurring effusive FIP. Leveraging single-cell RNA sequencing (scRNA-seq), multiplex immunofluorescence, and RNA *in situ* hybridization, we identified FIPV RNA and viral nucleocapsid protein in T and B lymphocytes, in addition to monocyte-derived cells. Notably, we detected subgenomic RNA within CD3^+^ T cells, consistent with active viral RNA synthesis. These findings challenge the long-standing view that FIPV infection is confined to monocyte/macrophages and instead reveal a broader tropism that includes key components of the adaptive immune system. By documenting *in situ* lymphocyte infection in a naturally occurring large-animal coronavirus model, this study lays the groundwork for re-examining immune dysregulation, viral persistence, and post-treatment relapse through a translational lens relevant to both veterinary and human medicine.

## 2. Materials and methods

### 2.1. Sample collection

Formalin fixed and paraffin embedded (FFPE) cat lymph node tissue samples in this study were obtained from archived specimens at the UC Davis William R. Pritchard Veterinary Medical Teaching Hospital (VMTH) Pathology laboratory. All samples were obtained from cats with naturally occurring effusive FIP that had succumbed to disease and were not treated with any antiviral drugs. The tissues were fixed in 10 % neutral buffered formalin for a minimum of 48 h and subsequently embedded in paraffin blocks. Sections measuring approximately 5 μm in thickness were cut from each lymph node tissue block and routinely processed for multiplex staining.

Additionally, 11 client owned cats with naturally occurring effusive FIP that presented to the VMTH between April 2022 and April 2023 were enrolled in a veterinary clinical trial as previously described (Wanakumjorn et al., 2025). All cat owners signed an informed consent form, and an Institutional Animal Care and Use Committee protocol (# 2232) of the University of California, Davis was approved. Briefly, all cats were etiologically diagnosed based on a combination of clinical presentation, imaging findings, laboratory results, and confirmatory detection of FCoV RNA in effusions via RT-PCR (Kennedy, 2020; Tasker et al., 2023). All cats were serotyped and determined to be serotype 1 (Murphy et al., 2024a). Cats were randomly assigned to either receive a combined antiviral (GS-441524) and mesenchymal stromal cell (MSC) therapy or antiviral with sham infusions. Ultrasound-guided aspirates of mesenteric lymph nodes were collected by a board-certified veterinary radiologist prior to initiation of therapy and at the completion of the study (12 weeks) and processed for scRNA-seq to assess immune cell composition and viral tropism at baseline.

Mesenteric lymph node aspirates were processed for scRNA-seq analysis exactly as previously described (Wanakumjorn et al., 2025). Briefly, aspirates were flushed with cold phosphate-buffered saline (PBS, GIBCO cat#10010023) supplemented with 2 % fetal bovine serum (FBS Atlanta Biologicals, cat# S11150). The cells were then centrifuged at 400 g for 10 min at 4°C, followed by the addition of Red Blood Cell (RBC) lysis buffer (eBioscience, cat# 00-4333-57). After a 3- minute incubation, cold media (Dulbecco’s Modified Eagle Medium/Nutrient Mixture F-12 (DMEM/F-12, ThermoFisher Scientific, cat# 11320033) supplemented with 10 % FBS) was added, and the samples were centrifuged at 200 g for 10 min at 4°C. The supernatant was removed, and the cell pellets were resuspended in 750 μl of Cell Prefixation Buffer (Parse Bioscience, cat# ECLC3501). Fixation was carried out using the Parse Bioscience Cell Fixation (v1 or v2) kit, and the samples were stored at −80°C until barcoding and library preparation were performed using the Evercode Whole Transcriptome kit (Parse Bioscience, ECWT3300).

### 2.2. Library sequencing and data analysis

Single-cell libraries were sequenced on an Illumina NovaSeq 6000 S2 100 flow cell with a targeted sequencing depth of 50,000 reads per cell. Basecalling was conducted using the Illumina NovaSeq 6000 RTA software (v3.4.4), and FASTQ files were generated using the bcl2fastq Conversion software (v2.20), which converts the BCL base call files produced by the sequencing instrument into FASTQ format. Data pre-processing and alignment were carried out using the SPLiT-seq pipeline provided by Parse Biosciences (split-pipe v1.0.3p), which processes raw FASTQ files into gene expression matrices. Specifically, raw sequencing reads were aligned to the feline (Felis_catus_9.0) and FIPV (EU186072 and DQ010921) reference genomes utilizing the STAR aligner (https://github.com/alexdobin/STAR), and gene expression was quantified based on raw counts.

### 2.3. scRNA-seq data management, analysis, and visualization

The scRNA-seq dataset was processed and analyzed using the Trailmaker^®^ community platform (https://scp.biomage.net/) provided by Parse Biosciences. Pre-filtered count matrices were uploaded for downstream analyses, and barcodes underwent a four-step filtration process to ensure data quality. Barcodes with fewer than 500 unique molecular identifiers (UMIs) were excluded. To remove low-quality cells, barcodes with mitochondrial read content exceeding 1 %—a marker of cell stress or death—were discarded. A robust linear model, implemented using the MASS R package (v7.3–56), was employed to detect outliers by establishing a relationship between the number of genes detected per barcode and the UMI count. Barcodes outside a tolerance interval of 1-alpha (where alpha is the inverse of the total droplets in the sample) were removed. Doublet detection was performed using the scDblFinder R package (v1.11.3), and barcodes with doublet scores greater than ~0.5 were excluded to maintain single-cell resolution. After filtering, each sample retained between 160 and 14,000 high-quality barcodes, which were then processed for integration and clustering.

To prepare the data for integration, log-normalization was applied, and the top 2000 highly variable genes were selected using the variance-stabilizing transformation (VST) method. Principal component analysis (PCA) was performed, with the top 40 principal components (PCs) accounting for 88.78 % of the total variance. These PCs were then used for batch effect correction with the Harmony R package. Cell clustering was conducted using the Louvain algorithm, implemented through the Seurat R package. Visualization was achieved using Uniform Manifold Approximation and Projection (UMAP) embedding, executed via Seurat’s UMAP wrapper. The resulting UMAP plots were manually annotated by comparing cluster-specific marker genes with known markers from relevant literature, supplemented by data from the Cellkb database (https://www.cellkb.com/). Finally, differential gene expression (DEG) analysis was performed for each cluster using the Wilcoxon rank-sum test from the presto R package. We report adjusted *p*-values using the Benjamini-Hochberg method to control for false discovery rate (FDR). Pathway enrichment analysis of DEGs was also conducted using standard hypergeometric testing, with significance defined as FDR < 0.05.

### 2.4. Immunofluorescence

FFPE lymph node tissue sections were deparaffinized prior to antigen retrieval using boiling commercially available buffer (DAKO Target Antigen Retrieval Solution, pH 6.1, cat# S2375), followed by blocking using donkey serum at room temperature for 1 h. Sections were then incubated with primary antibodies, including anti-CD20 (Polyclonal Rabbit anti-human; 1:100; Invitrogen, cat#16701), anti-CD3 (CA17.2A12 rat anti-canine; 1:10; Leukocyte Antigen Biology Laboratory), anti-IBA1 (Polyclonal Rabbit anti-IBA1;1:100; FUJIFILM, cat#019-19741) and anti-FCoV nucleocapsid (monoclonal Mouse anti-Feline corona virus; 1:100; BIORAD, cat# MCA2194).

After overnight incubation at 4°C, the slides were washed with Tris buffered saline with tween 20 (TBST) and stained with secondary antibodies: Alexa Fluor 488 donkey anti-rabbit (Invitrogen, cat# A21206), Alexa Fluor 647 donkey anti-rat (Invitrogen, cat# A48272), and Alexa Fluor 594 donkey anti-mouse (Invitrogen, cat# A21203) at room temperature for 1 h. Cell nuclei were visualized using DAPI nuclear counterstain (Invitrogen, cat# D1306), and slides were mounted using ProLong Gold Antifade Mountant (Thermo Fisher Scientific, cat# P36934).

### 2.5. Combined in situ RNA hybridization and Immunofluorescence

To detect FIPV RNA within cells expressing CD3, CD20, and IBA1, a combined RNAscope/ BaseScope assay (for FIPV RNA detection) and immunofluorescence assay (for CD3, CD20, and IBA1 detection) was utilized, enabling the simultaneous detection of FIPV-RNA hybridized probes and fluorophore-labeled protein targets. FFPE lymph node tissue sections were deparaffinized and hybridized with the RNAscope V-FIP-M-C1 probe (ACD, cat# 1591971-C1)/ BaseScope BA-V-FIPV-Leader-M-Junc-C1probe (ACD, cat# 1591981-C1). Control probes, including the RNAscope Positive Control Probe PPIB-C1/POLR2A-C2/UBC-C3 (ACD, cat# 24261 A) and the RNAscope Negative Control Probe DapB (ACD, cat# 320871), BaseScope Positive Control Probe BA-Fc-PPIB-3zz-st-C1 (ACD, cat# 1134211-C1) and BaseScope Negative Control Probe BA-DapB-3ZZ (ACD, cat# 701011-C2) were included, and the assay was conducted according to the manufacturer’s protocol. TSA Vivid Fluorophores 650 (ACD, cat#323273) was added according to the manufacturer’s protocol.

Following the final staining step using RNAscope Multiplex Fluorescent Detection reagent/BaseScope Detection reagents v2-RED kit, tissue sections were rinsed and incubated in a blocking solution containing 15 % donkey serum and 1 % FcR blocking reagent (Miltenyi Biotec, cat# 130-059-901) for 1 h. Primary antibodies, including anti-CD3, CD20 and IBA1, were applied and incubated overnight at 4°C. Secondary antibodies— Alexa Fluor 488 donkey anti-rabbit, Alexa Fluor 647 donkey anti-rat, and Alexa Fluor 594 donkey anti-mouse were applied at a 1:1000 dilution for 1.5 h, followed by DAPI staining for nuclear visualization.

### 2.6. Confocal microscopy and image analysis

High-resolution, Z-stacked images of feline lymph node were acquired using a Leica TCS SP8 STED 3X confocal microscope equipped with a 20 × /0.75 oil objective (HC PL APO CS2) and 100 × /1.4 oil objective (HC PL APO CS2). Imaging was performed with 0.2 μm optical slices at a resolution of 1024 × 1024 pixels. The raw.lif image files were subsequently processed using the CMLE algorithm in Huygens Professional software (Scientific Volume Imaging, http://svi.nl) for deconvolution.

## 3. Results

We enrolled 11 cats with spontaneous effusive FIP to a clinical trial, as previously described (Wanakumjorn et al., 2025). All 11 cats were determined to be infected with a serotype 1 virus (Murphy et al., 2024a). Clinical and demographic information regarding this cat population is provided in Table 1.

**Fig. 1.**
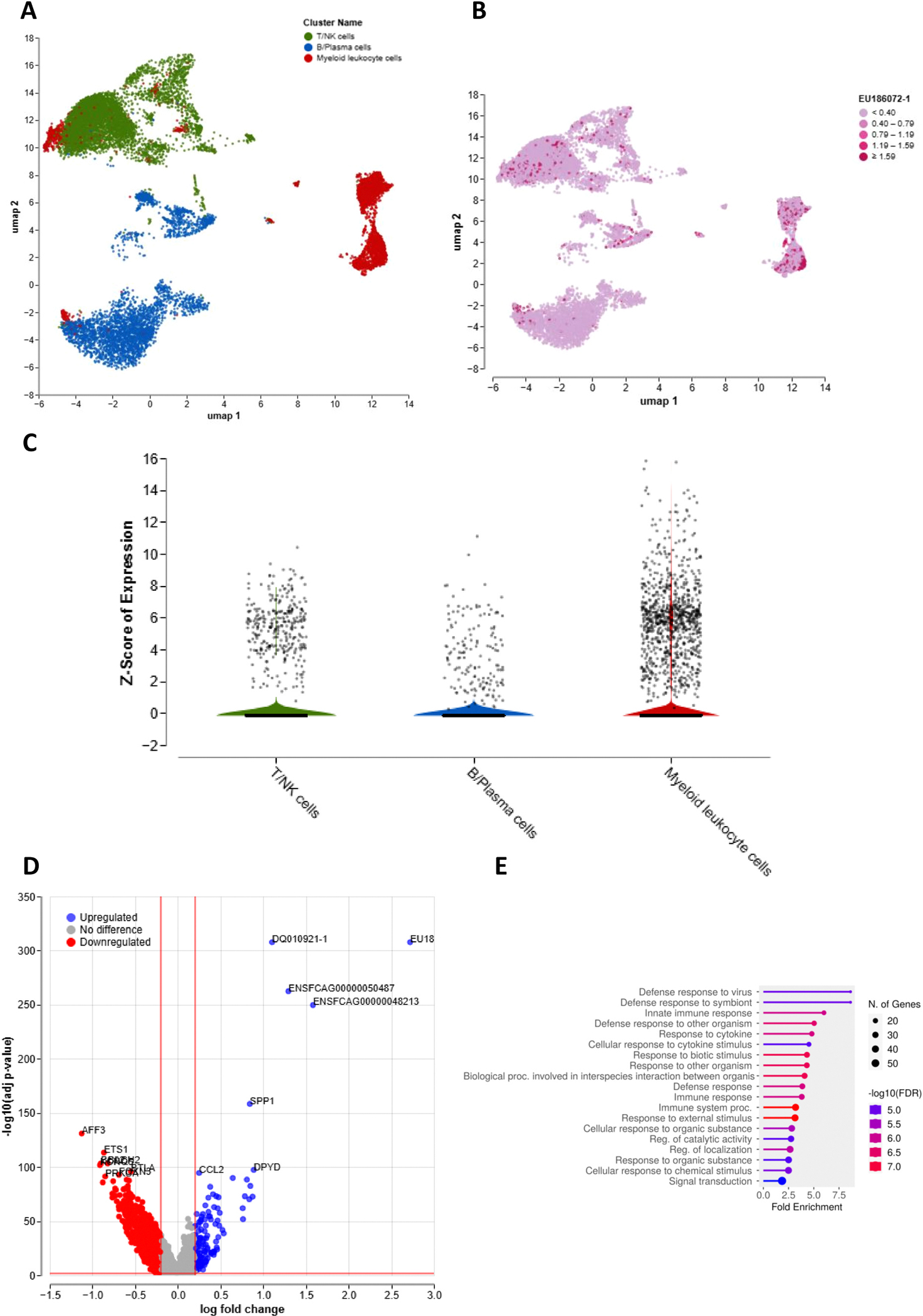
FIPV RNA is detected via scRNA-seq in myeloid and lymphoid immune cell subsets in lymph nodes of cats with naturally occurring effusive FIP. (A) UMAP visualization of 14,029 high-quality single-cell transcriptomes from mesenteric lymph node aspirates of cats with effusive FIP (pre-treatment samples), showing three major immune cell compartments: T/NK cells, B/plasma cells, and myeloid leukocytes. (B) UMAP plot of the same dataset highlighting cells with detectable FIPV RNA (red), demonstrating that viral RNA–positive cells are present across all three major immune compartments. (C) Violin plots displaying the distribution of FIPV RNA reads across each immune cell compartment, confirming that while myeloid cells contain the highest viral load, both T/NK and B/plasma cell clusters also harbor FIPV RNA sequences. (D) Volcano plot showing differentially expressed genes in FIPV RNA–positive versus FIPV RNA–negative cells. Upregulated antiviral and innate immune response genes, including DDX60, MX1, RNF213, and RBM47, are prominently represented. (E) Pathway enrichment analysis of differentially expressed genes in FIPV RNA–positive cells, demonstrating significant enrichment of pathways related to defense response to virus, innate immune response, and interferon signaling.

After the cats were enrolled in the study and prior to any therapeutic intervention, ultrasound guided mesenteric lymph node aspirates were obtained for scRNA-seq. A second lymph aspirate was obtained after 12 weeks of treatment. Following quality control filtering, we obtained high-resolution transcriptomic profiles from a total of 50,793 (visit #1: 14,029; visit #2: 36,764) high-quality cells (Fig. 1A). Dimensionality reduction and clustering analysis revealed three major immune compartments: T/NK cells, B/plasma cells, and myeloid leukocytes. Notably, at the time of trial enrollment 1401 cells contained FIPV RNA sequences, enabling direct interrogation of *in vivo* viral tropism (Fig. 1B). As expected, FIPV RNA was most abundant in myeloid cells. However, FIPV RNA was also detected in T/NK and B/plasma cell clusters. Specifically, we found viral sequences in 5.35 % of T cell/NK cells, 3.36 % of B/plasma cells and 21 % of myeloid leukocytes. Moreover, mean Z scores of expression for the viral sequences were −0.5193, −0.6335 and 1.153 in T/NK cells, B/plasma cells and Myeloid leukocytes, respectively, suggesting a broader range of viral tropism than previously appreciated (Fig. 1C).

Differential gene expression and pathway enrichment analyses were performed between all of the FIPV+ cells and the FIPV− cells (Fig. 1D). This analysis was designed to determine whether the presence of FIPV RNA within cells was associated with distinct, virus-induced transcriptional responses, thereby confirming that these sequences reflected biologically meaningful infection rather than incidental uptake of non-functional viral fragments or a bioinformatic artifact. Infected cells showed increased transcription of antiviral and type I/III interferon-stimulated genes, including DDX60, MX1, RNF213, and RBM47. Pathway enrichment analysis further revealed activation of pathways involved in defense response to virus and innate immune response (FDR<0.05, Fig. 1E). These findings indicate that FIPV RNA sequences are present in myeloid cells as well as in a wide range of lymphocyte subsets and that FIPV+ cells have activated transcriptional machineries that reflect innate immune response to viral infection.

To validate and spatially localize viral infection within lymphoid tissues, we performed multiplex immunofluorescence staining of FFPE mesenteric lymph nodes from cats with effusive FIP that were not treated and succumbed to the disease. We screened mesenteric lymph nodes from 10 cats for FCoV nucleocapsid protein abundance, as determined by immunofluorescence. We chose to continue our investigation using FFPE mesenteric lymph node tissues from 2 cats. Clinical and histologic findings in these cats are summarized in Table 2.

**Fig. 2.**
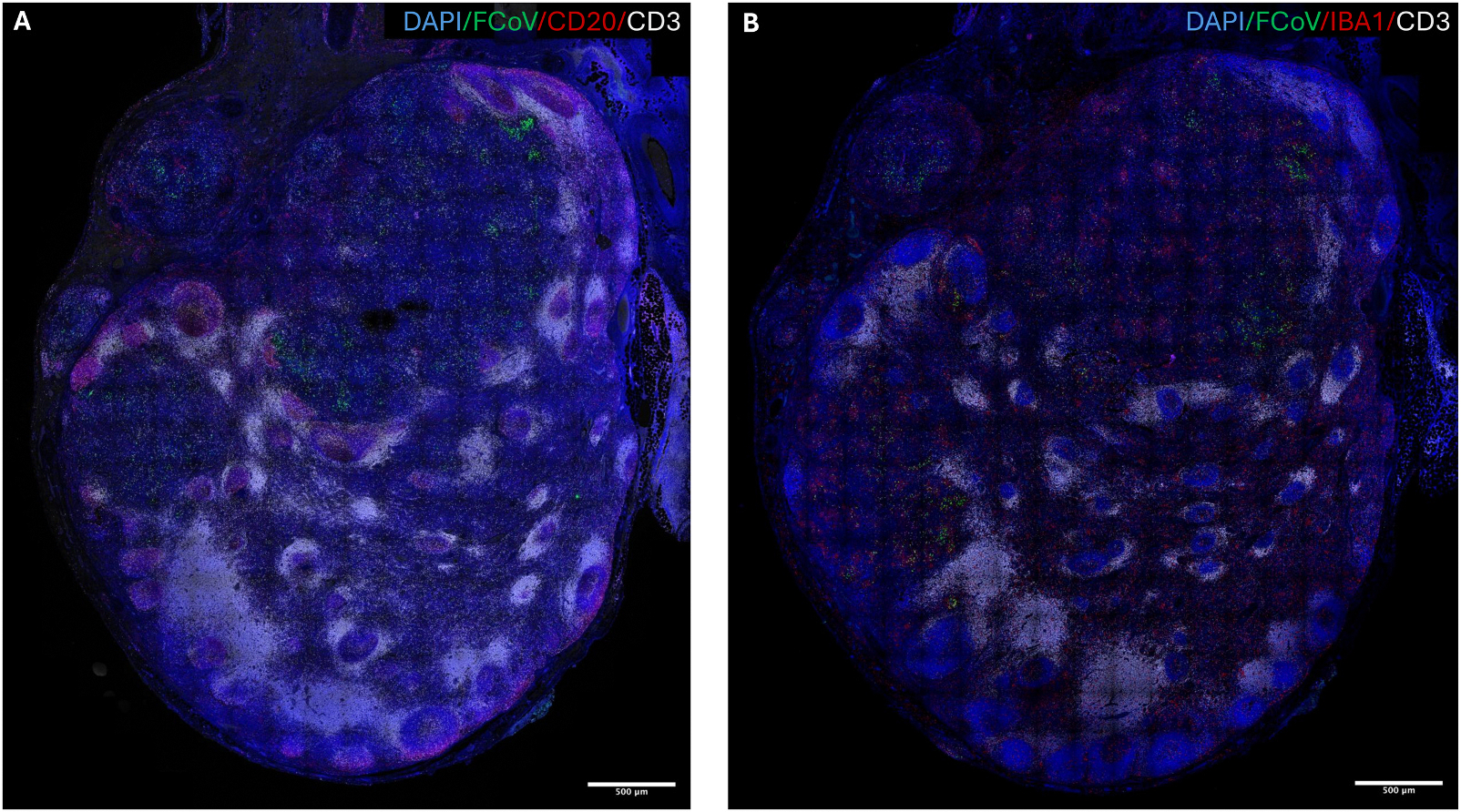
FCoV antigen distribution and disruption of lymph node architecture in cats with effusive FIP. (A) Low-magnification confocal image of a mesenteric lymph node section stained with DAPI (blue, nuclei), FCoV nucleocapsid protein (green), CD20 (red, B cell marker), and CD3 (grey, T cell marker). Normal follicular architecture is disrupted and replaced by expanded cortical regions and granulomatous infiltrates. Scale bar = 500 μm. (B) Low-magnification confocal image of a mesenteric lymph node section stained with DAPI (blue), FCoV nucleocapsid protein (green), IBA1 (red, macrophage marker), and CD3 (grey). FCoV nucleocapsid antigen is enriched within IBA1^+^ macrophage-rich granulomas, along the nodal capsule. Scale bar = 500 μm.

MsLN were stained with antibodies against FCoV nucleocapsid protein (Green, Fig. 2A and B) and lineage-specific markers: CD3 (Grey, Fig. 2A and B),CD20 (Red, Fig. 2A) and IBA-1 (Red, Fig. 2B). Findings revealed that normal follicular architecture was disrupted and replaced by multifocal IBA1^+^ granulomatous infiltrates. Consistent with prior reports, FCoV signal was enriched in these granulomas and along the nodal capsule (Fig. 2).

**Fig. 3.**
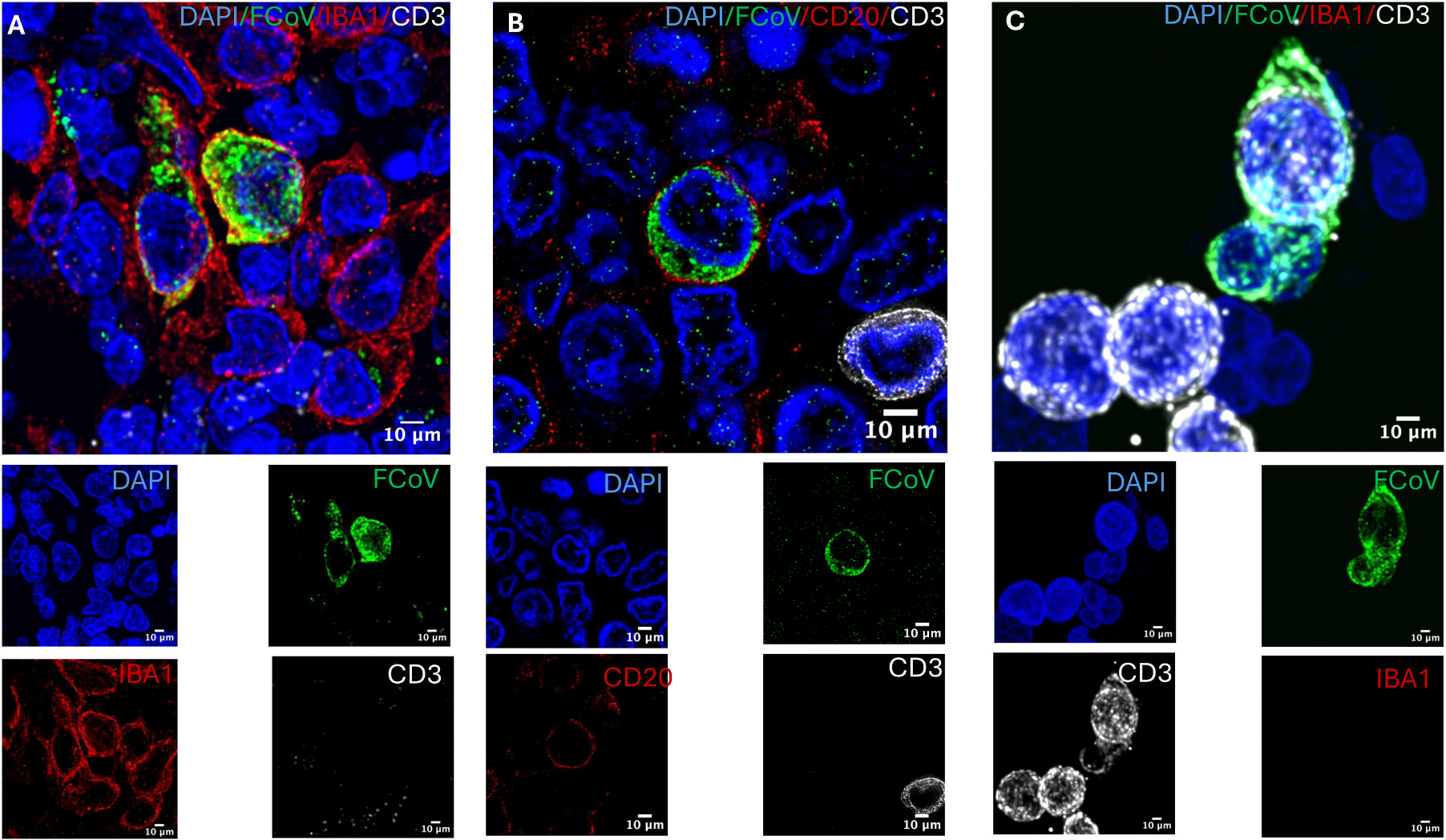
FCoV nucleocapsid protein localizes to IBA1^+^ macrophages, CD20^+^ B cells, and CD3^+^ T cells in mesenteric lymph nodes of cats with effusive FIP. High-magnification (100 ×) confocal images of FFPE mesenteric lymph node sections stained with DAPI (blue, nuclei), FCoV nucleocapsid protein (green), and lineage-specific markers (red). (A) FCoV nucleocapsid protein is present within IBA1^+^ macrophages. (B) FCoV nucleocapsid protein detected in CD20^+^ B cells. (C) FCoV nucleocapsid protein detected in CD3^+^ T cells. Scale bar = 20 μm.

High-resolution confocal imaging at 100 × magnification showed clear FCoV signal within IBA1^+^ histiocytes (Fig. 3A). Further supporting our transcriptomic data, FCoV signal was also rarely observed in CD20^+^ B cells (Fig. 3B) and CD3^+^ T cells (Fig. 3C). The latter findings demonstrate the presence of viral protein (nucleocapsid) within monocyte/macrophages as well as lymphocyte subsets.

**Fig. 4.**
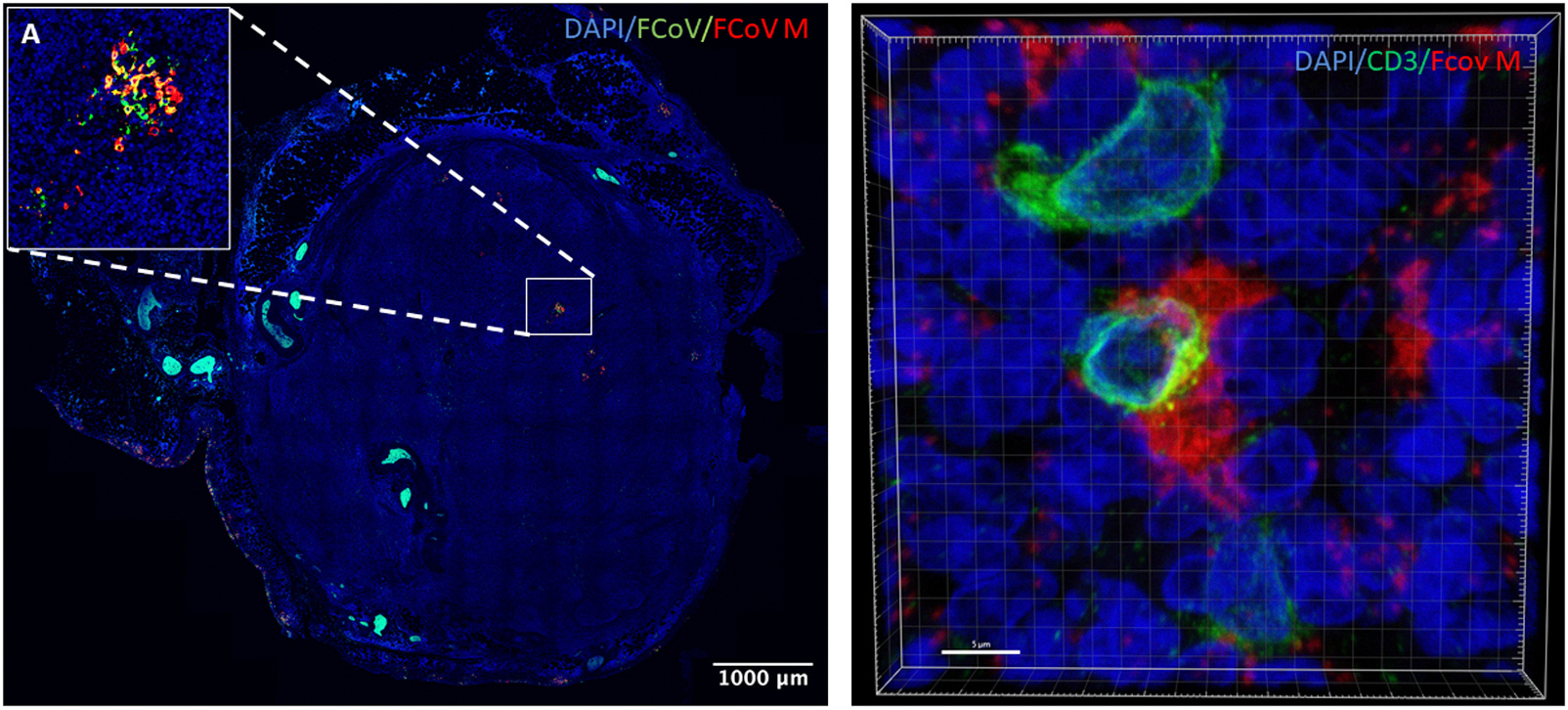
FIPV M gene RNA localizes to T cells in lymph nodes of cats with effusive FIP. High-magnification (100 ×) confocal images of FFPE mesenteric lymph node sections subjected to combined RNAscope *in situ* hybridization for FIPV M gene RNA (green) and immunofluorescence for lineage-specific markers. (A) FIPV M gene RNA signal overlaps with FCoV nucleocapsid protein signal, validating the RNAscope probe specificity. Scale bar = 1000 μm.(B) FIPV M gene RNA is present within CD3^+^ T cells (red), indicating the presence of viral RNA within T lymphocytes. Scale bar = 20 μm.

To determine whether viral replication occurred in these lymphoid subsets, we conducted a combined RNA fluorescence *in situ* hybridization (FISH) and immunofluorescence assay. We first validated an RNAscope probe that targets the viral M gene by multiplexing it with the anti-FCoV antibody. Z stack confocal microscopy studies demonstrate that antigen and RNA sequences partially spatially overlap, as indicated by the orange color (Fig. 4A). We further multiplexed the RNAscope probe with the cell identity antibody markers. Unfortunately, the only antibody that successfully multiplexed with the RNAscope probe was the T cell marker CD3. However, confocal microscopy studies demonstrate viral M gene RNA FISH signal within CD3 positive T cells, though infrequently (Fig. 4B). These studies demonstrated that CD3 positive T cells not only contain viral proteins, but also viral RNA. Attempts to multiplex M gene RNA FISH with CD20 and IBA1 immunofluorescence were unsuccessful.

**Fig. 5.**
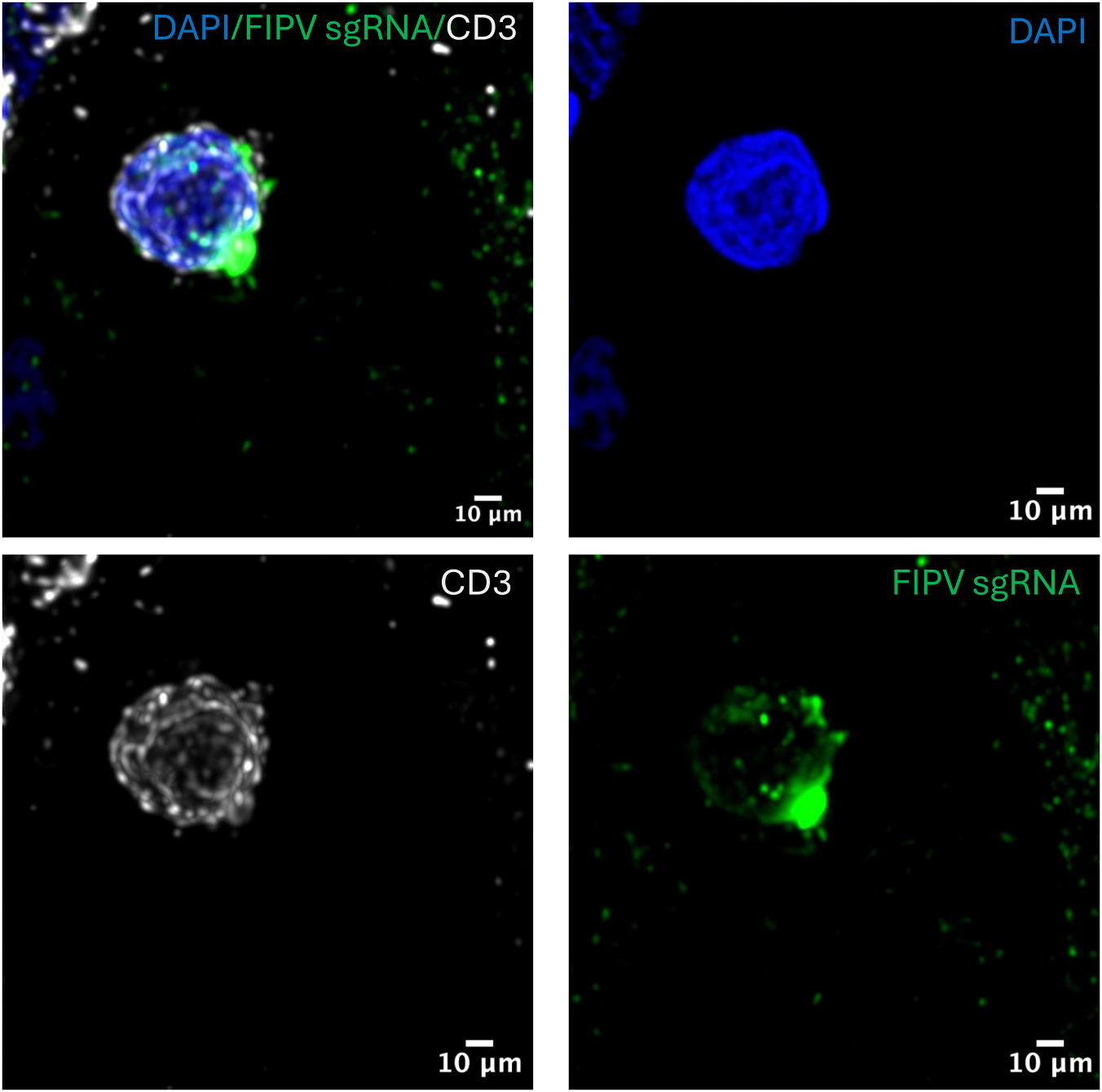
FIPV sgRNA localizes to T cells in mesenteric lymph nodes of cats with effusive FIP. High-magnification (100 ×) confocal image of a mesenteric lymph node section stained with DAPI (blue, nuclei), FIPV sgRNA (sgRNA; green), and CD3 (grey, T cell marker). FIPV sgRNA signal is present within CD3^+^ T cells, indicating that FIPV actively replicates within T lymphocytes *in vivo*. Scale bar = 10 μm.

To further determine if lymphocytes are permissive to FIPV genomic replication, we sought to multiplex cell identity markers with BaseScope assay for FIPV subgenomic RNA (sgRNA) sequences. sgRNA is a positive-sense, 5’-capped and 3’-polyadenylated RNA molecule synthesized during coronaviral replication that contains a common 5’ leader sequence joined to downstream open reading frames (ORFs). These sgRNAs serve as messenger RNAs (mRNAs) for the expression of structural and accessory proteins encoded toward the 3’ end of the viral genome (V’kovski et al., 2021). Z stacked confocal microscopy studies demonstrated FIPV sgRNA signal (green, Fig. 5) within CD3^+^ T cells (Grey, Fig. 5), indicating viral genomic replication in T cells within lymphoid tissues (Fig. 5). Attempts to multiplex the sgRNA BaseScope with CD20 and IBA1 immunofluorescence were unsuccessful.

**Fig. 6.**
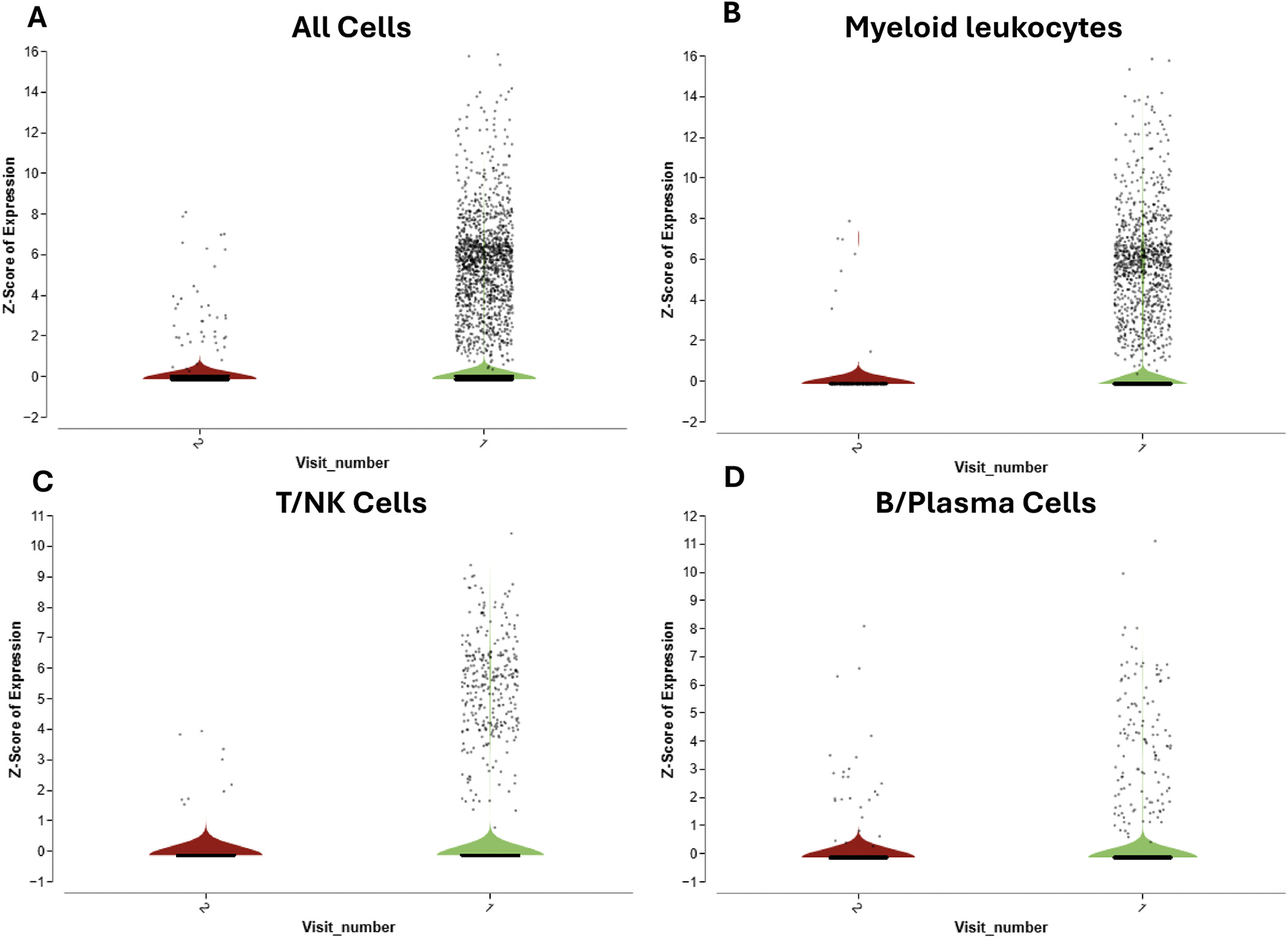
FIPV RNA persists at low levels in all major immune cell compartments following antiviral treatment. Violin plots showing the distribution of FIPV RNA reads across (A) all cells, (B) myeloid leukocytes, (C) T/NK cells, and (D) B/plasma cells in mesenteric lymph node aspirates collected pre (visit #1) and post (visit #2) treatment. While the overall number of FIPV RNA–positive cells is markedly reduced following therapy, residual viral RNA is still detectable at low levels in all major immune compartments.

Finally, while all of the FIP cats that were enrolled in the clinical trial recovered and did not show clinical signs of disease or relapse at the end of the study, scRNA-seq analysis of mesenteric lymph nodes post treatment (i.e. visit #2) indicated that FIPV RNA was rarely identified (Fig. 6). All 3 major cell identity categories (i.e. myeloid leukocytes, T/NK and B/Plasma cells) had cells with detectable FIPV RNA.

Together, these findings expand the current understanding of FIPV tropism, demonstrating that viral infection and viral genomic replication can occur in lymphoid cells beyond the traditionally accepted monocyte/macrophage compartment.

## 4. Discussion

This study provides the first direct *in situ* evidence that FIPV invades and undergoes genomic replication within T lymphocytes in cats with naturally occurring effusive FIP. Using single-cell transcriptomics, multiplex immunofluorescence, and BaseScope RNA *in situ* hybridization, we demonstrate that FIPV RNA and nucleocapsid protein are detectable in phenotypically defined lymphocytes, with subgenomic viral RNA, a marker of active genomic replication, localized specifically to CD3^+^ T cells. While we did not observe full evidence of productive infection (i.e., virion assembly and egress), the presence of both viral structural proteins and viral replicative intermediates within lymphocytes suggest that lymphocytes may be permissive to FIPV infection. These findings revise the prevailing paradigm of FIP pathogenesis by expanding the known viral cellular tropism to include adaptive immune cells, and raise important questions about the implications of lymphocyte infection for immune dysregulation, viral persistence, pathogenesis, and treatment response.

Identification of productive viral infection requires more than the presence of viral particles or RNA; it necessitates evidence of viral entry into a host cell, translation of viral proteins, replication of the viral genome, assembly of progeny virions, and culminates in the egress of infectious virions. The detection of sgRNA in particular is considered a hallmark of coronaviral genomic replication, as these transcripts are synthesized only within infected cells during the transcription of structural and accessory genes by the viral replicase complex (V’kovski et al., 2021; Hornyak et al., 2012). While our study does not provide direct evidence of full virion assembly or release, the detection of viral nucleocapsid protein and sgRNA within phenotypically defined T lymphocytes strongly suggests that FIPV can enter and replicate the viral genome within these cells.

A major challenge in elucidating the molecular determinants of FIPV cellular tropism lies in the limited ability to model serotype 1 infection *in vitro*. FIPV exists in two serotypes, 1 and 2, that differ in spike protein sequence and receptor usage (Herrewegh et al., 1998). While serotype 2 strains can be propagated in cell culture and efficiently infect primary feline macrophages via fAPN, serotype 1 viruses, which account for the overwhelming majority of naturally occurring FIP cases (Benetka et al., 2004; Kummrow et al., 2005), do not use fAPN and fail to replicate reliably in primary cells or established cell lines (Hohdatsu et al., 1998; Dye et al., 2007). Consequently, mechanistic studies of serotype 1 viral infection and host range have been hampered by the absence of robust *in vitro* culture systems. All cats in our veterinary clinical trial were confirmed to be infected with serotype 1 FIPV (Murphy et al., 2024b), underscoring the translational relevance of our findings. While our study is the first one to show viral antigen and RNA within host T cells, it does not provide more in-depth mechanistic insight regarding host cell entry. In this context, *in situ* approaches such as single-cell transcriptomics, multiplexed immunofluorescence, and RNA *in situ* hybridization offer a critical alternative, enabling direct assessment of viral tropism in the native tissue microenvironment and overcoming key limitations of traditional *in vitro* models.

Historically, the cellular tropism of FIPV has been based on early foundational ultrastructural and immunohistochemical studies, which have strongly implicated monocytes and macrophages as the primary target cells. Seminal electron microscopy EM) investigations, including those by Ward (1970) and Horzinek et al. (1977), identified coronavirus-like particles in histiocytes within granulomatous lesions of experimentally infected cats. However, these studies relied exclusively on morphological criteria to identify cell types, a subjective method that is vulnerable to misclassification, especially in inflamed or necrotic tissues. Moreover, uncommon events, such as FIPV-infected lymphocytes, may be readily missed when scanning a slide with EM. Subsequent immunohistochemical (IHC) studies, such as those by Tammer et al. (1995) and Kipar et al. (1998) demonstrated FIPV antigen in macrophage-like cells using immunohistochemistry, but these early assays did not incorporate multiplexed cell lineage markers, making it difficult to definitely determine cell-host identity.

Our study overcomes these limitations by combining scRNA-seq, multiplex immunofluorescence, and RNAscope *in situ* hybridization to precisely map FIPV RNA, protein, genomic RNA and subgenomic transcripts to phenotypically defined cell types within lymphoid tissues derived from cats with naturally occurring FIPV serotype 1 infection. By detecting viral RNA and replication intermediates specifically in CD3^+^ T cells, confirmed via high-resolution imaging, we provide direct evidence that FIPV infects and replicates its genome within a broader range of immune cells than previously recognized. This integrative, multi-modal approach enables us to distinguish true infection from nonspecific staining or phagocytosis of viral debris, thereby significantly advancing the understanding of FIPV pathogenesis.

Our findings have important implications for the pathogenesis of acute disease but also relapsing and chronic forms of FIP. The detection of viral genomic RNA and sgRNA transcripts in lymphocytes suggests that these long-lived, migratory immune cells may serve as reservoirs of persistent infection, a concept that is supported by our data and is well established in human coronavirus infections such as SARS-CoV-2 and MERS-CoV (Pontelli et al., 2022; Brunetti et al., 2023; Chu et al., 2016; Shen et al., 2022). In this context, infected lymphocytes could facilitate viral persistence beyond clinical remission, providing a mechanistic basis for the recrudescence of disease or chronic immune dysregulation observed in some cats following antiviral treatment (Taylor et al., 2023; Murphy et al., 2024a; Zwicklbauer et al., 2023; Trsar et al., 2025; Gokalsing et al., 2025). Furthermore, direct infection of T and B cells introduces a novel dimension to FIP pathogenesis by implicating the possibility of adaptive immune cell dysfunction, not just macrophage-driven inflammation, as a driver of immune collapse. This finding may contribute to the profound lymphopenia, impaired immune memory, and poor viral control seen in affected cats. Together, these findings call for a broader therapeutic approach that targets not only viral replication but also immune restoration to prevent relapse and support durable remission.

Despite the novel insights gained from this study, several limitations should be acknowledged. First, functional validation of productive infection is constrained by the absence of reliable *in vitro* culture systems for FIPV serotype 1 isolates, particularly in primary feline immune cells. Second, the scRNA-seq analysis was limited by the modest number of high-quality cells recovered from aspirates, which may have restricted our ability to detect rare cell populations or subtle phenotypic transitions. Specifically, while neutrophils and fibroblasts play a significant role in pyogranulomatous inflammation and may further be targeted by the FIP virus, they were present at very low abundance in our scRNA-seq data set, and did not form distinct, high-quality clusters after quality control filtering. As a result, we could not perform a meaningful or statistically robust comparison of FIPV RNA abundance within these populations. Third, we were unable to definitively distinguish between cytotoxic versus helper T cells, or between naïve, memory, and plasma B cell subsets in our imaging or transcriptomic validation assays. This precluded a more granular analysis of which adaptive immune cell types are most susceptible to infection or functionally impaired. Finally, as our study focused exclusively on cats with effusive FIP, future studies will be necessary to determine whether similar patterns of infection and immune disruption occur in the ‘dry’ form of the disease.

Taken together, our findings confirm the paradigm that FIPV serotype 1 infects monocyte/macrophages in naturally occurring effusive FIP and further provide compelling *in situ* evidence that FIPV invades T and B lymphocytes and undergoes genomic replication within T cells. This potentially expanded tropism revises long-standing assumptions about FIPV pathogenesis and highlights adaptive immune cells as direct viral targets, a feature increasingly implicated in other coronavirus-associated syndromes, including severe COVID-19, MIS-C, and Long COVID. By integrating single-cell transcriptomics with spatially resolved validation, this study establishes a new comparative framework for dissecting coronavirus-immune system interactions *in vivo*. Beyond veterinary relevance, FIP in the domestic cat represents a naturally occurring animal model that enables direct access to lymphoid tissues during natural severe coronavirus infection, offering unique translational opportunities to investigate immune cell–mediated viral persistence, immunopathology, and treatment failure. As antiviral therapies continue to improve clinical outcomes, a deeper understanding of how coronaviruses invade and modulate adaptive immune compartments will be critical for developing interventions that promote long-term immune recovery and prevent relapse. Future studies should focus on delineating the molecular mechanisms of lymphocyte infection, assessing the functional impact on immune competence, and determining whether these potentially infected populations serve as latent or relapsing reservoirs. Such insights will not only refine the therapeutic approach to FIP but also inform broader models of coronavirus immunopathogenesis across species.

## Figures and Tables

**Table 1:** Clinical and demographic data of cats included in the prospective clinical trial.

Case #	Breed	Sex	Age	Presenting complaint	Diagnostic workup

1	DMH	M	8MO	Inappetence and lethargy	CBC, Serum biochemistry, abdominal US, abdominal fluid analysis, Fluid FeCV PCR
2	DSH	MC	6MO	Lethargy, weight loss, decreased appetite/drinking	CBC, Serum biochemistry, abdominal US, abdominal fluid analysis, Fluid FeCV PCR
3	DSH	MC	9MO	Lethargy, weight loss, decreased appetite/drinking	CBC, Serum biochemistry, abdominal US, abdominal fluid analysis, Fluid FeCV PCR
4	Bengal	M	4MO	Less active and has been losing weight	CBC, Serum biochemistry, abdominal US, abdominal fluid analysis, Fluid FeCV PCR
5	DSH	FS	6MO	Lethargy, weight loss, decreased appetite/drinking	CBC, Serum biochemistry, abdominal US, abdominal fluid analysis, Fluid FeCV PCR
6	DMH	MC	7MO	Lethargy and inappetence and fever	CBC, Serum biochemistry, abdominal US, abdominal fluid analysis, Fluid FeCV PCR
7	Scottish fold	MC	10MO	Lethargy, hiding, and decreased appetite	CBC, Serum biochemistry, abdominal US, abdominal fluid analysis, Fluid FeCV PCR
8	DSH	MC	4MO	Decreased appetite, energy and diarrhea	CBC, Serum biochemistry, abdominal US, abdominal fluid analysis, Fluid FeCV PCR
9	Ragdoll	MC	4MO	Distended abdomen and lethargy	CBC, Serum biochemistry, abdominal US, abdominal fluid analysis, Fluid FeCV PCR
10	DSH	MC	1YO	Icterus of the inner pinna, weight loss, decreased playing, and a progressively distended abdomen.	CBC, Serum biochemistry, abdominal US, abdominal fluid analysis, Fluid FeCV PCR
11	DMH	M	8MO	Decreased appetite and lethargy	CBC, Serum biochemistry, abdominal US, abdominal fluid analysis, Fluid FeCV PCR

DMH - Domestic medium hair, DSH - Domestic short hair, M - Male, MC - Male castrated, FS - Female spayed, MO - Months old, YO - Years old, CBC - Complete blood count, US - ultrasound.

**Table 2:** Clinical, demographic and pathologic data of cats from which FFPE tissues were obtained.

Case #	Breed	Sex	Age	Presenting complaint	Diagnostic workup	Serotype	Pathologic findings

1	DSH	FS	4YO	Fever of Unknown origin, weight loss and lethargy	CBC, Serum biochemistry, abdominal US	ND	Effusive FIP characterized by widespread pyogranulomatous and lymphoplasmacytic inflammation affecting the duodenum, liver, pancreas, lymph nodes, pleura, and lateral brain ventricle. Additional findings included myeloid and thymic hyperplasia, mesothelial hyperplasia, and mild ocular inflammation. Immunohistochemistry confirmed FIPV antigen within MsLN, hepatic and intestinal pyogranulomas and variably in the ventricular ependyma. Overall, the lesions supported a diagnosis of FIP with extensive serosal and parenchymal involvement.
2	Bengal	FS	9MO	Weight loss, lethargy and fever of unknown origin	CBC, Serum biochemistry, abdominal US	Type 1	Effusive FIP with icterus and abundant fibrinonecrotizing, pyogranulomatous serositis involving the omentum, liver, spleen, pancreas, kidneys, and intestinal serosa, together with granulomatous lymphadenitis and severe duodenitis with Peyer’s patch depletion. There was marked hepatitis, pancreatitis, and splenitis, accompanied by yellow effusion and widespread serosal plaques. Immunohistochemistry demonstrated strong coronavirus antigen across multiple organs, confirming systemic FIPV-associated pyogranulomatous inflammation.

DSH - Domestic short hair, FS - Female spayed, MO - Months old, YO - Years old, CBC - Complete blood count, ND - Not determined, US - ultrasound.

## Data Availability

The data underlying this article will be shared on reasonable request to the corresponding author.
